# Supplementary material for: Contactless probing of polycrystalline methane hydrate at pore scale suggests weaker tensile properties than thought
Source: Nat Commun. 2020 Jul 6;11:3379. doi: 10.1038/s41467-020-16628-4 (PMC7338411; doi:10.1038/s41467-020-16628-4)
Supplement: Supplementary file 1 — Supplementary Information [file 41467_2020_16628_MOESM1_ESM.pdf]

Supplementary Information for
Contactless probing of polycrystalline methane hydrate at pore scale suggests weaker tensile properties than thought

by Atig *et al.*

Supplementary Notes

Supplementary Note 1: Materials and methods

Materials

We use fused silica capillaries as model siliceous pores (Vitrotubes, CMScientific, as received). Tubes used here were 10 cm long, with internal and external diameters 200 and 330 μm. Water was deionized (Purelab classic system, electrical resistivity 18 MΩcm^{-1}). The methane was 99.9995 % grade (Linde). DASPI (trans-4-[4-(Dimethylamino)styryl]-1-methylpyridinium iodide) was obtained from Aldrich.

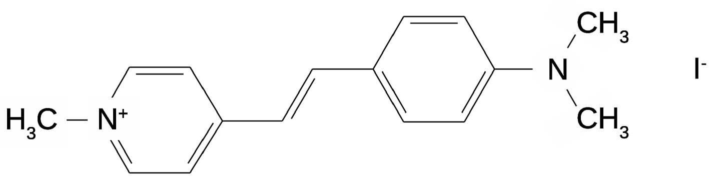

Supplementary Figure 1 | Structural formula of the rigidochrome dye DASPI.

Sample preparation

N.B. Glass capillaries are strong but fragile! In order to accommodate expansion, operator clumsiness *etc*, hold them only at one point end and allow free movement otherwise. Wear eye protection when handling capillaries– accidental splinters of glass may fly. As with all experiments at high pressures, potential users should first consult their local laboratory safety advisor.

A capillary open at both ends is dipped in deionized water until the water rises inside by 10–25 mm. The wet end is sealed with a micro-torch. In order to facilitate formation of the hydrate and to avoid formation of ice, all air must be carefully excluded. We remove bubbles by centrifuging the capillary before use.

Pressure and temperature control

An ISCO DM65 syringe pump controls the gas pressure to the inlet of a three-way needle valve (TopIndustrie). The capillary is glued with epoxy glue (Loctite) in a 1/16" steel tube connected to one outlet of the valve. Cyanoacrylate glues are not recommended here since they do not cure reproducibly in the confined space between the steel and the glass, and further, vapours of the uncured glue may be flushed to the sample space, where they polymerise with the water. The other outlet and the valve on the pump provide for thorough flushing of the line and sample with methane at the start of an experiment. The capillary is inserted in a cooling and heating stage with observation windows, mostly a Linkam Cap500 with Linksys temperature control software. The capillary can be moved in the two horizontal directions in the temperature controller stage. Temperature control is provided by a silver block and a cover with a 50 mm long, 1 mm wide, 0.7 mm deep rectangular channel, which hosts the capillary. A central hole through both parts provides optical access. The temperature is controlled by a combination of electrical heating and a a flow of cold nitrogen, driven by the Lynksys32 software. The temperature is folded into the video recordings with a home-made Qt aplication.[1] Temperature homogeneity is improved by the presence of an IR filter between the light source and the microscope condenser, to prevent local heating of the observation zone. Temperature stability is estimated to be better than ± 0.1 K in the temperature range of interest, and, important here, steady rates of change in the range from 0.1 to 50 K/minute can also be imposed.

Microscopy

Most observations are made with the Linkam capillary holder on an Olympus BX50 upright microscope stand with a x10 extra-long working distance objective (Olympus) and a Ueye UI 3360 camera run at 1 frame per second. Fluorescence and high resolution microscopies are performed on a Nikon Ti-Eclipse inverted microscope stand, with a custom thermostat

block (Étincelage, Serres-Castet, France) with a Peltier cooler. This thermostat is set in a home-made housing with a dry nitrogen flow to exclude condensation. The position of the capillary in the field of view of a x20 objective (Nikon, FI Plan Fluor ELWD 20XC) may be adjusted from outside the housing with an XYZ micro-translation stage (parts from Thorlabs). Data are recorded with an Orca 4.0 sCMOS camera (Hamamatsu) at up to 20 frames per second.

Temperature cycles

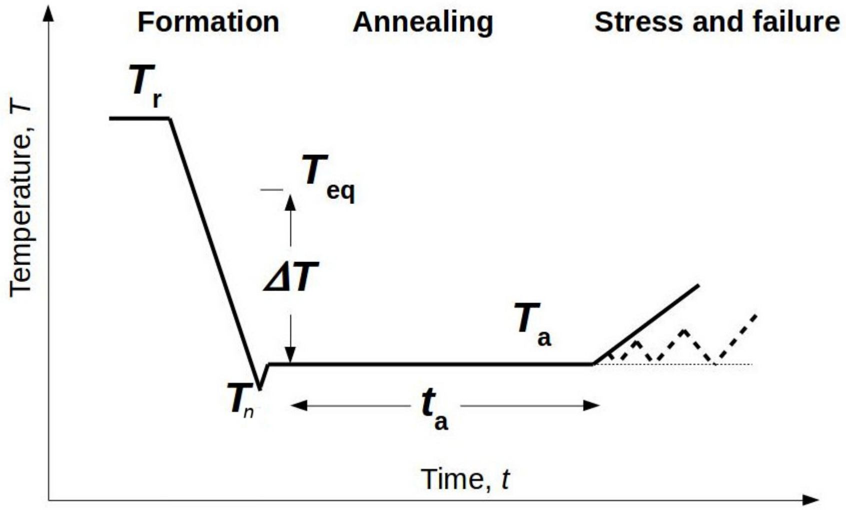

Supplementary Figure 2 | Steps in the formation and stressing of the hydrate shell at constant gas pressure. Following saturation of the water with methane at high pressure ($\approx$ 150 bar) and room temperature, T_r, the sample is quenched at constant gas pressure, until quasi-simultaneous initiation of the hydrate as a cap on the meniscus, as micro-crystals in the water and as a polycrystalline halo riding the glass on a film of water under the gas, at $T_n \approx -23\,°C$. After annealing at temperature $T_a \geq T_n$ (hours), the sample is warmed at constant rate (over a period of $\approx$ 100 s, solid line), or cycled to increasing temperatures (dashed line), until failure of the hydrate. $\Delta T = T_{eq} - T_a$ is the supercooling, with T_{eq} the dissociation temperature of the hydrate.

The capillary is left 15 h at room temperature and 15 MPa of methane, to saturate the water column with the gas prior to an experimental run. The pressure is held constant until the end of a run.

A home-made Qt application folds the temperature and cooling rate from the temperature controller into the video recordings for later reference. Supplementary Figure 2 shows the sequence of steps to form the hydrate:

1. The system is cooled from ambient temperature at 5 K/min until methane hydrate forms on the meniscus, usually in the temperature range $-27 < T_\mathrm{n} < -20\,^\circ\mathrm{C}$.

2. Water at such temperatures is metastable. In order to avoid ice, the temperature is raised at 5 K/min to the annealing temperature, T_a, at which the shell is allowed to lengthen, thicken and change texture for a defined period.

3. We stress the hydrate shell at approximately constant stress rate, by increasing temperature at a constant rate, gas pressure remaining constant. (At the experimental temperature, raising the temperature causes contraction). The relation between the thermal pressure differential and the temperature excursion is detailed below in Supplementary Note 3.

Supplementary Note 2 : Image processing

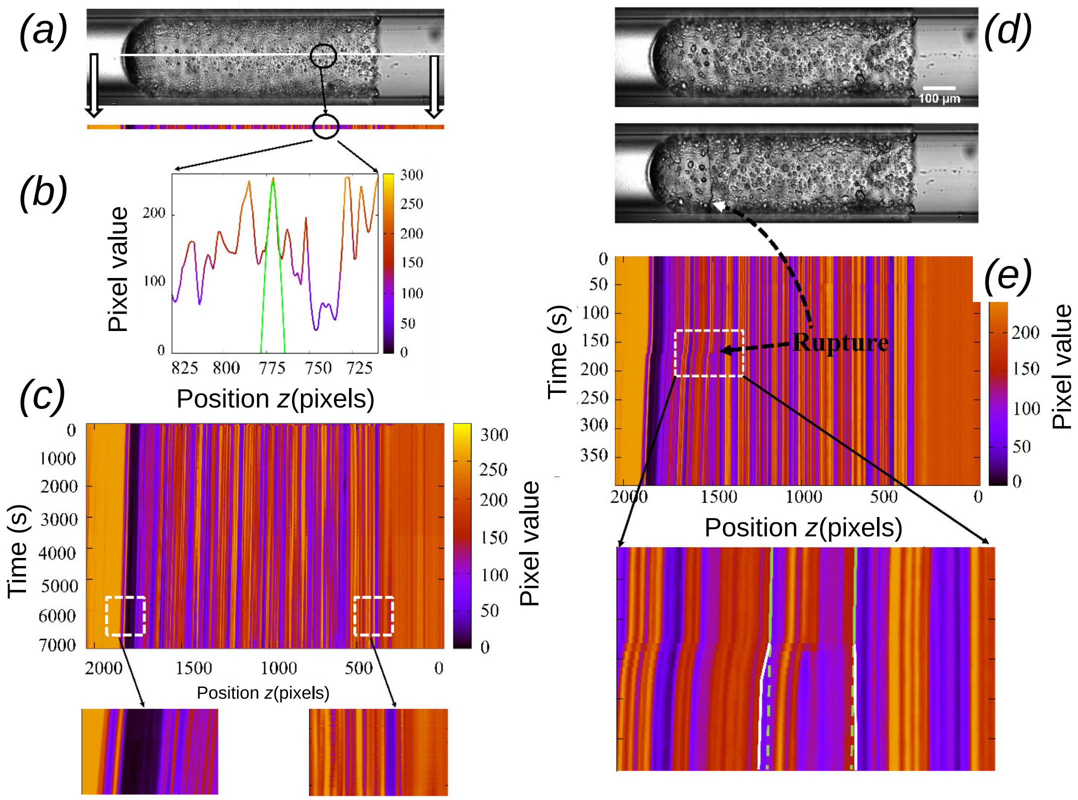

Supplementary Figure 3 | Image processing to extract: *(i)* the elongation in the halo, during annealing *(a–c)* on the left, or *(ii)* the axial strain during tensile tests (*d-e*,right). *(a)* A particular line near the axis of the capillary is chosen and its pixel values are exported as a (text) vector from all frames of the video; *(b)* Bright features in the halo (peaks in the line) are identified in the first frame and a parabola is fitted to a window of points (typically ≈ 5) in the immediate neighbourhood of each peak (green curve); the parabola is updated from frame to frame, using as initial fit parameters the values from the previous frame. The peak of the parabola defines the instantaneous position of the feature; *(c)* Assembling the intensity-colour coded lines from all frames as a map, $I(z,t)$, provides an overview of the dynamics of the halo, where z is the abscissa (pixel) and t, the time (frame number) is the ordinate. The enlargements show elongation or its absence near the cap and the halo tip (which is immobile, adhering to the glass); *(d)* Images from a tensile test, taken at the start and just after failure, which is highlighted by the fork in a bright line around $t = 175\,\mathrm{s}$, close to pixel

1600, see *(e)*. The enlargement shows hydrate ahead of the failure snapping to the left; that to the right hardly retracts, due to plastic deformation. Halo annealed for $t_a = 7\,\mathrm{h}$ at $\Delta T = 40.3\,\mathrm{K}$.

Digital image correlation is a common method of extracting a strain field from video recordings of flat samples, using intrinsic or added markers in the material.[2] Here, aberration introduced by the curved glass means that only a narrow longitudinal strip of the halo is sharp at any focus of the objective, so we apply a home-made algorithm. Soon after breaking out of the cap, the halo is circularly symmetrical around the capillary axis. Its elongation is therefore that of any line parallel to the axis. We choose a line in sharp focus in the videos, passing close to the optical axis, on the near side with respect to the objective. The pixel values of the line, I(z,t), are extracted from all frames of the video, where z is the ordinate (pixel) and t the time (frame number). For an overview of the elongation of the halo we plot the lines as a colour coded map of I(z,t), see Supplementary Figure 3*(c,e)*. Permanent features of the halo, such as crystallites give rise to dark or bright bands, whose inclination off the vertical axis indicates the local rate of deformation. Curved bands show acceleration, *e.g.* when plastic deformation sets in. The failure of the halo is easier to locate accurately in this representation than by just replaying the videos, since it gives rise to an obvious fork in the bands, see Supplementary Figure 3*(e)*.

Starting from the first frame, we identify bright features of the halo from the local maxima of the intensity, $I(z, t = 0)$. We define the position of the features by a least squares fit of a parabola to points in a window around each maximum (typically ≈ 5 points). The green curve in Supplementary Figure 3b is an example. The movements of all the features are tracked by updating the fits from frame to frame, using the parameters of the parabolas in the previous frame as starting values. The whole process, automated in a combination of *bash* and *gnuplot*[3] scripts, typically provides coordinates of ≈ 50 features over thousands of frames, from which we calculate the local strain, *e.g.* in figure of the main text.

The same image processing is used for monitoring the growth of the hydrate shell during the annealing period, Supplementary Figure 3*(a-c)* and during a tensile test leading to shell rupture (part *d-e*). These two processes occur on very different time scales: hours in the former case, *vs.* minutes in the latter. Shell rupture usually occurs along a circle transverse to the exerted force, see figure, part *(d)*, and the rupture process can thus be investigated using the above method. Brittle rupture has in fact a clear signature, see Supplementary Figure 3*(e)* and its caption.

Supplementary Note 3: Corrections to the simple temperature-stress relation

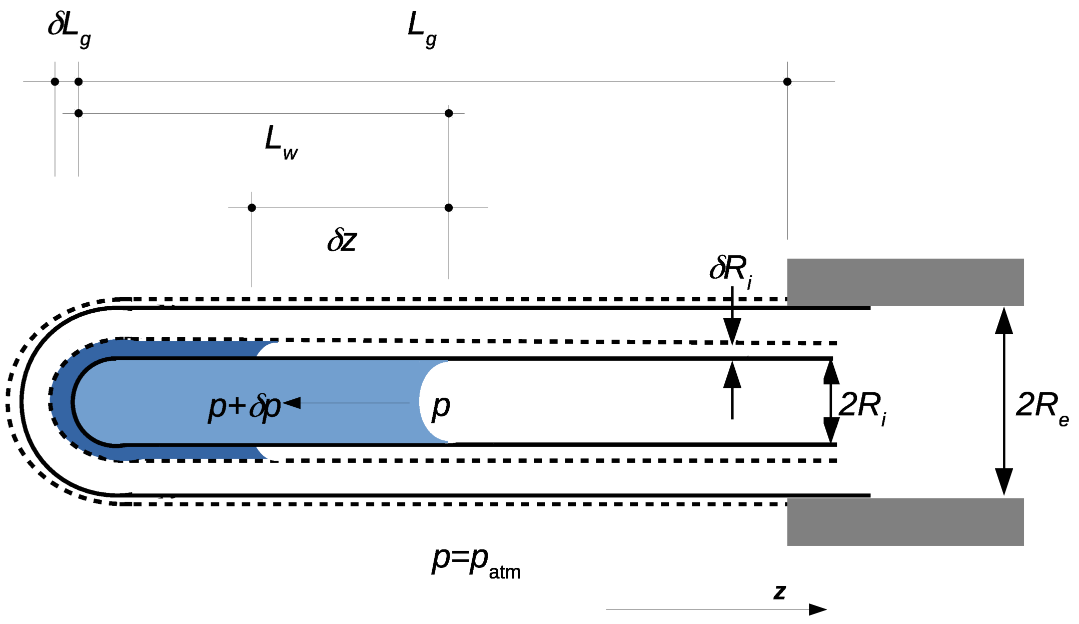

Supplementary Figure 4 | Schematic of the compression of a liquid and the deformation of the glass on application of pressure jump δp to the gas above a column of liquid in a capillary glued in the steel pressure tubing. Light/dark blue: initial and final states of the liquid. The initial and final dimensions are indicated by solid and dashed lines.

This section discusses corrections to the simple expression for the tensile stress, eq. (3) of the main text. First, consider a change of pressure, from p to $p + \delta p$ possibly at the same time as a change of temperature δT, in a capillary of length L_g containing a column of liquid, for example water (w), of initial length L_w. Let the initial internal and external radii of the capillary be R_i and R_e, *cf.* Supplementary Figure 4. In independent runs without forming the hydrate, the apparent or measured compressibility, β_m, or the apparent coefficient of volumetric thermal expansion, α_m are deduced from the relative change of volume of the aqueous phase, equated with the displacement of the meniscus, δz. This amounts to ignoring change of the contact angle, actually negligible here, and change of shape and size of the capillary, corrected shortly below:

$$\frac{\delta V_\mathrm{w}}{V_\mathrm{w}} \approx \frac{\delta z}{L_\mathrm{w}} \quad . \tag{1}$$

In this equation, compressibility or expansivity are related to non-vanishing δz, while in the presence of the hydrate crust enclosing the water, $\delta z = 0$, so the constraint $\delta V_\mathrm{w} = 0$ relates δT and δp.

Equation 1 assumes the capillary is perfectly rigid. In fact, its thermal expansion and its elasticity, though too small to detect directly, contribute to the adjustment of the position of the gas:water interface. (The sealed end of the capillary is well outside the field of view.) The displacement of the end of the capillary is due to the stretching or contraction of both the glass and the steel high pressure tube, as far as the anchor point in the three-way valve. However, the Young's modulus of glass is about three times smaller than that of steel and temperature variations affect only the glass. Therefore, we neglect the latter contribution.

The initial volume of liquid is

$$V_\mathrm{w} \approx \pi R_\mathrm{i}^2 L_\mathrm{w} \quad , \tag{2}$$

where L_w is measured before inserting the capillary in the sample holder. To first order of

infinitesimals, the change of volume of the liquid corresponds to the (algebraic) changes of *(i)* the length of the column of liquid, $\delta L_{\mathrm{w}} = \delta z - \delta L_{\mathrm{g}}$ and *(ii)* the change of the diameter, $2\delta R_{\mathrm{i}}$.

$$\begin{aligned} \delta V_{\mathrm{w}} &= -\beta_{\mathrm{w}} V_{\mathrm{w}} \delta p + \alpha_{\mathrm{w}} V_{\mathrm{w}} \delta T \quad , &(3) \\ &\approx \pi R_{\mathrm{i}}^2 \delta L_{\mathrm{w}} + 2\pi R_{\mathrm{i}} \delta R_{\mathrm{i}} L_{\mathrm{w}} \quad , \\ \delta V_{\mathrm{w}} &= \pi R_{\mathrm{i}}^2 \left(\delta z - \delta L_{\mathrm{g}}\right) + 2\pi R_{\mathrm{i}} \delta R_{\mathrm{i}} L_{\mathrm{w}} \quad . &(4) \end{aligned}$$

In these relations, β_{w} and α_{w} are the true isothermal compressibility and isobaric coefficient of volumetric expansion. Note the presence of both L_{g} and L_{w} in the last relation, since the change of L_{w} is not measurable, but estimated from $\delta L_{\mathrm{w}} = \delta z - \delta L_{\mathrm{g}}$. Strictly speaking, L_{g} and R_{i} are coupled, but the capillary is very long, here $L_{\mathrm{g}} \approx 80\,\mathrm{mm}$ compared to R_{i}, $R_{\mathrm{e}} \approx 0.3\,\mathrm{mm}$, so we may estimate δL_{g} and δR_{i} separately. The change of the bore of an infinite cylindrical pipe in response to a change of the internal pressure may be found for example in Landau & Lifshitz.[4] Adding the contribution of the thermal expansion of the glass (isobaric coefficient of linear expansion α_{g}):

$$\delta R_{\mathrm{i}} = \frac{\delta p R_{\mathrm{i}}^2}{R_{\mathrm{e}}^2 - R_{\mathrm{i}}^2} \frac{1+\sigma}{Y} \left[R_{\mathrm{i}} \left(1 - 2\sigma\right) + \frac{R_{\mathrm{e}}^2}{R_{\mathrm{i}}} \right] + \alpha_{\mathrm{g}} \delta T R_{\mathrm{i}} \quad , \tag{5}$$

where Y and σ are the Young's modulus and the Poisson ratio of the glass. This expression may be written in terms of the bulk and shear moduli of the glass, K and G and the dimensionless aspect ratio $\eta = R_{\mathrm{i}}/R_{\mathrm{e}} < 1$:

$$\frac{2\delta R_{\mathrm{i}}}{R_{\mathrm{i}}} = \frac{1}{1-\eta^2} \frac{\delta p}{G} \left[1 + \eta^2 \frac{3G}{G + 3K} \right] + 2\alpha_{\mathrm{g}} \delta T \quad . \tag{6}$$

The change of pressure exerts a force, F, on the sealed end of the capillary, that con-

tributes to the change of length δL_{g}. After projection on the capillary axis,

$$F = -\pi R_{\mathrm{i}}^2 \delta p \quad .$$

The traction is applied over the section of the capillary wall,

$$A = \pi \left(R_{\mathrm{e}}^2 - R_{\mathrm{i}}^2 \right) \quad ,$$

Including the contribution of thermal expansion:

$$\begin{aligned} \delta L_{\mathrm{g}}/L_{\mathrm{g}} &= \frac{1}{Y}\frac{F}{A} - \alpha_{\mathrm{g}}\delta T \quad , \\ &= \frac{-\eta^2}{1-\eta^2}\frac{\delta p}{Y} - \alpha_{\mathrm{g}}\delta T \quad , && (7) \\ &= \frac{-\eta^2}{1-\eta^2}\frac{K+G/3}{3KG}\delta p - \alpha_{\mathrm{g}}\delta T \quad . && (8) \end{aligned}$$

The sign in front of α_{g} is negative since thermal expansion causes the end of the capillary to move left in Supplementary Figure 4 (when $\alpha_{\mathrm{g}}\delta T > 0$). Combining equations (2- 4) and (8), we have the corrected form of eq. (3) of the main text:

$$\boxed{-\beta_{\mathrm{m}}\delta p + \alpha_{\mathrm{m}}\delta T = \frac{\delta z}{L_{\mathrm{w}}}} \quad , \qquad (9)$$

where we distinguish longitudinal and radial contributions of the elasticity and thermal expansion of the glass to the measured α_{m} and β_{m}:

$$\begin{aligned} \beta_{\mathrm{m}} &= \beta_{\mathrm{w}} + \delta\beta^{\mathrm{long}}(L_{\mathrm{g}}, L_{\mathrm{w}}) + \delta\beta^{\mathrm{rad}} \quad , && (10) \\ \delta\beta^{\mathrm{long}}(L_{\mathrm{g}}, L_{\mathrm{w}}) &= \frac{K+G/3}{3KG}\frac{L_{\mathrm{g}}}{L_{\mathrm{w}}}\frac{\eta^2}{1-\eta^2} \quad , && (11) \\ \delta\beta^{\mathrm{rad}} &= \frac{1}{1-\eta^2}\frac{1}{G}\left[1 + \eta^2\frac{3G}{G+3K}\right] \quad , && (12) \end{aligned}$$

and

$$\alpha_{\mathrm{m}} = \alpha_{\mathrm{w}} - \delta\alpha^{\mathrm{long}}(L_{\mathrm{g}}, L_{\mathrm{w}}) - \delta\alpha^{\mathrm{rad}} \quad , \tag{13}$$

$$\delta\alpha^{\mathrm{long}}(L_{\mathrm{g}}, L_{\mathrm{w}}) = \frac{L_{\mathrm{g}}}{L_{\mathrm{w}}}\alpha_{\mathrm{g}} \quad , \tag{14}$$

$$\delta\alpha^{\mathrm{rad}} = 2\alpha_{\mathrm{g}} \quad . \tag{15}$$

Examples

- **True isothermal compressibility of the liquid** : Set $\delta T = 0$ in eq. (9) and recall that

$$\left[\frac{\delta V_{\mathrm{w}}}{V_{\mathrm{w}}}\right]_{\mathrm{apparent}} = \frac{\delta z}{L_{\mathrm{w}}} = -\beta_{\mathrm{m}}\delta p \quad . \tag{16}$$

Then from (10), the corrected estimate of the compressibility is

$$\beta_{\mathrm{w}} = \beta_{\mathrm{w}} - \left\{\delta\beta^{\mathrm{long}}(L_{\mathrm{g}}, L_{\mathrm{w}}) + \delta\beta^{\mathrm{rad}}\right\} \quad . \tag{17}$$

Supplementary Figure 5*(a)* shows the isothermal compressibility of water pre-saturated with methane at 15 MPa at room temperature ($T_{\mathrm{r}} = 20\,^{\circ}\mathrm{C}$) before and after correction, prior to forming the hydrate. We estimate the correction using the following values:

  - $K \approx 35\,\mathrm{GPa}$, $G \approx 29\,\mathrm{GPa}$, typical of borosilicate glass,[5,6]
  - $\eta = 100/165 \approx 0.61$,
  - $L_{\mathrm{w}} = 20\mathrm{mm}$,
  - $L_{\mathrm{g}} = 80\mathrm{mm}$.

Then, despite the multiplicative effect of the length of the capillary, it is the radial dilation which is the larger contribution: $\delta\beta^{\mathrm{long}}(L_{\mathrm{g}}, L_{\mathrm{w}}) \approx 3.4 \times 10^{-2}\ \mathrm{GPa}^{-1}$, $\delta\beta^{\mathrm{rad}} \approx 7.2 \times 10^{-2}\ \mathrm{GPa}^{-1}$, and the total correction is 15 – 20 % for T between -23.5 °C

and -5 °C.

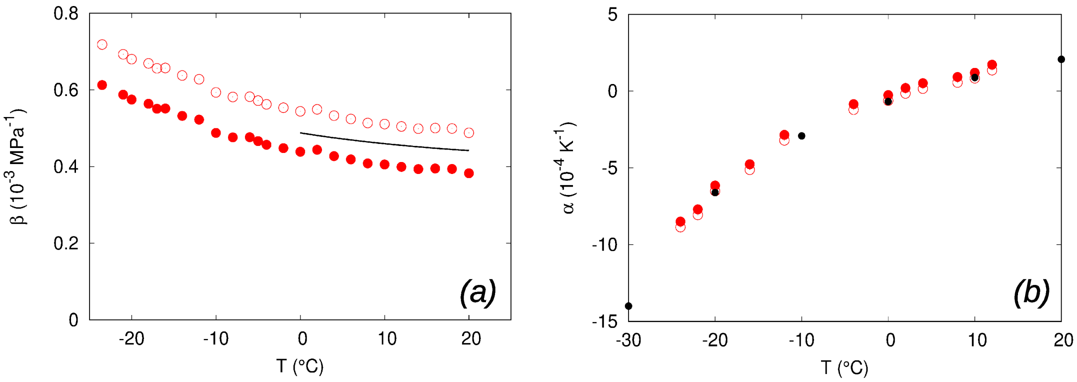

Supplementary Figure 5 | *In situ* measurement of the isothermal compressibility and the isobaric thermal (volume) expansion coefficients of water at 15 MPa, pre-saturated ≈ 15 h at room temperature, $T_r = 20\,°C$, with methane at the same pressure. *(a)* Isothermal compressibilities. Open symbols: apparent compressibility as measured by the displacement of the meniscus, solid symbols: after correction for the elasticity of the glass capillary. Solid line: pure water at 15 MPa, from ref.;[7] *(b)* Coefficient of isobaric volumetric thermal expansion of methane-saturated water (large symbols, same conditions as *(a)*), before (open) and after correction (solid). Small black symbols: for pure water at atmospheric pressure, from Kell.[8]

- **True isobaric thermal expansivity of the liquid** : Set $\delta p = 0$ in eq. (9) and use

$$\left[\frac{\delta V_w}{V_w}\right]_{\text{apparent}} = \frac{\delta z}{L_w} = \alpha_m \delta T \quad . \tag{18}$$

Then from (13),

$$\alpha_w = \alpha_m + \left(\delta\alpha^{\text{long}}(L_g, L_w) + \delta\alpha^{\text{rad}}\right) \quad , \tag{19}$$

which reduces to the expected $\alpha_w = \alpha_m + 3\alpha_g$ when $L_g = L_w$. Supplementary Figure 5*(b)* shows the isobaric volumetric thermal expansivity of the methane saturated water (*cf.* above) before and after correction. We use the same values of L_g and L_w,

and the linear expansivity of glass[9] $\alpha_g \approx 6 \times 10^{-6}\ \mathrm{K}^{-1}$. The longitudinal contribution dominates here, being two thirds of the total correction of $3.6 \times 10^{-5}\ \mathrm{K}^{-1}$. The total correction is however very small, *cf.* Supplementary Figure 5*(b)*.

- **Thermally induced pressure change behind the frozen hydrate shell** : Two changes are necessary in eq. (9). First, set $\delta z = 0$ because the hydrate shell replacing the meniscus adheres to the glass *via* its tip. Secondly, only the expansion of the length of glass between the hydrate cap and the closed end of the capillary need be taken into account to find the new volume of the vessel containing the liquid, so use $L_g = L_w$ and $L_g/L_w = 1$, implying smaller corrections. Thus

$$\delta p = \frac{\tilde{\alpha}_m}{\tilde{\beta}_m} \delta T \tag{20}$$

in which

$$\tilde{\beta}_m = \beta_w + \delta\beta^{\text{long}}(L_w, L_w) + \delta\beta^{\text{rad}} \tag{21}$$

and

$$\tilde{\alpha}_m = \alpha_w - \delta\alpha^{\text{long}}(L_w, L_w) - \delta\alpha^{\text{rad}} \quad . \tag{22}$$

The corrected form of equation for the stress in the hydrate shell, eq. (3) of the main text, is:

$$\boxed{\delta\sigma_a(t) \approx \frac{\tilde{\alpha}_m}{\tilde{\beta}_m} \frac{R}{2w} \delta T(t)} \quad , \tag{23}$$

in which the numerator and denominator may be found, once α_w and β_w are known, from eqs. (17) and (19), and $L_g = L_w$. The values of $\delta\beta^{\text{long}}$ and $\delta\alpha^{\text{long}}$ are now four times smaller than above, whereas the radial contributions are unchanged. Of course, strictly speaking, the fluid behind the shell is no longer the initially methane-saturated water, because formation of hydrate needles and their subsequent partial

dissolution (see main text) influence the concentration of dissolved gas. Furthermore the fluid contains a small solid fraction, the hydrate needles. However, Supplementary Figure 5*(a - b)* show that the dissolved methane has only a small effect on the properties. For example, β_w of the actual methane-depleted water behind the hydrate shell must lie between the solid line and the solid symbols in part *(a)*. Therefore, we continue to use the values β_w and α_w determined before formation of the hydrate, but with $L_g = L_w$ for computing the corrected values $\tilde{\alpha}_m$ and $\tilde{\beta}_m$.

Supplementary Note 4: A simple model of diffusion limited thickening of the hydrate shell

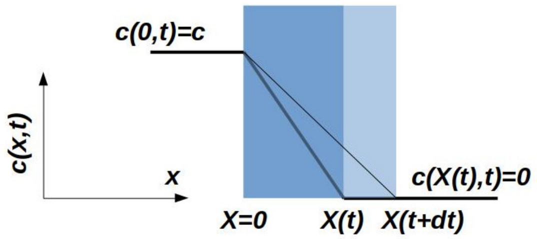

Supplementary Figure 6 | A simple model of the thickening of the hydrate shell by crystal growth limited by diffusion through the shell itself (blue shaded region). $c(x,t)$ is the concentration of the faster diffusing component of the shell, which has thickness $X(t)$ at time t.

The fact that micrographs show the cap and the halo all down its length to have comparable thicknesses at sufficiently long times, suggests that thickening is limited by transport of either the guest or the host across the shell. The strong supercooling in all our experiments is in favour of fast crystallization. Consider therefore a simple model in which one of the phases, in practice water, diffuses from its reservoir, through the existing shell to reach the side rich in the other phase, where crystallization is instantaneous. Let $X(t)$ be the thickness of the shell at time t ($X(t=0)=0$), and let $c(x,t)$ be the concentration in a plane parallel to the interface, at abscissa x at time t. The reservoir maintains the concentration constant on the left side of the shell in Supplementary Figure 6, so $c(x=0,t)=C$. Fast

crystallization, mopping up the mobile phase, maintains the condition $c(X(t), t) = 0$ at the opposite, growing face. Fick's second law reads

$$\frac{\partial c(x,t)}{\partial t} = D\frac{\partial^2 c(x,t)}{\partial x^2} \quad , \tag{24}$$

with the flux at abscissa x:

$$J(x,t) = -D\frac{\partial c(x,t)}{\partial x} \quad . \tag{25}$$

Assuming a quasi-steady state due to the invariant boundary conditions, $\partial c(x,t)/\partial t = 0$, from which (24) yields a linear concentration profile across the interface,

$$c(x,t) = C[X(t) - x]/X(t) \quad , 0 \le x \le X(t) \quad . \tag{26}$$

The linear profile implies a uniform flux in the second equation: $J(x,t) = DC/X(t)$. But the rate of crystallization is proportional to the flux at the surface

$$\frac{dX(t)}{dt} \propto J(x = X(t), t) = DC/X(t) \quad ,$$

whence

$$X(t)dX(t) \propto DCdt \tag{27}$$

and

$$X^2(t) \propto t \quad . \tag{28}$$

Supplementary Figures

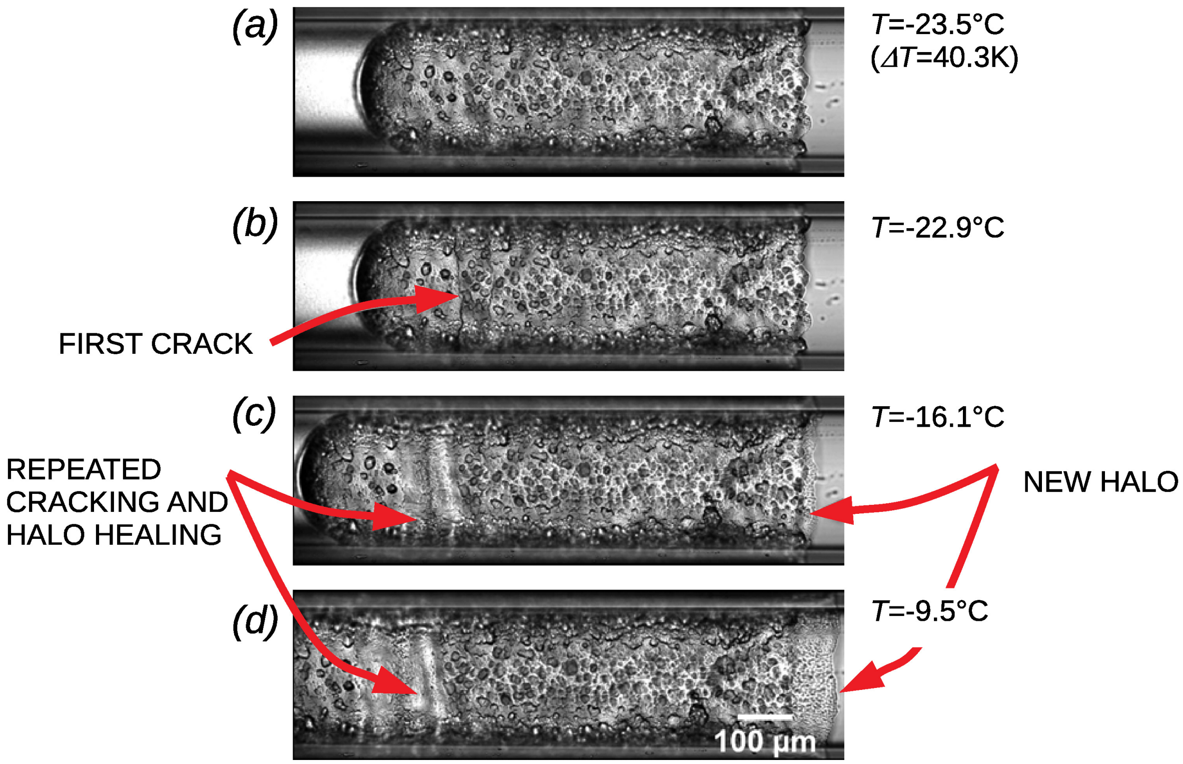

Supplementary Figure 7 | An example of healing after failure of the halo, with formation of a second halo riding on the first : *(a)* The halo at 15 MPa gas pressure, shortly before failure; *(b)* The moment of first failure (dark line near cap); *(c)* The cap snaps to the left, water invades the gap and overruns the older halo on the inside (gas side), forming fresh, finely textured hydrate; *(c – d)* The second halo continues spreading down the capillary, overrunning the tip of the first. As the temperature continues to rise, ($0.2\,\mathrm{K\,min^{-1}}$) the new halo repeatedly ruptures close to the cap. See also Supplementary Video 1. The initial supercooling was $\Delta T = 40.3$ K and in this example, the temperature ramp is pursued towards room temperature at the end of an experiment.

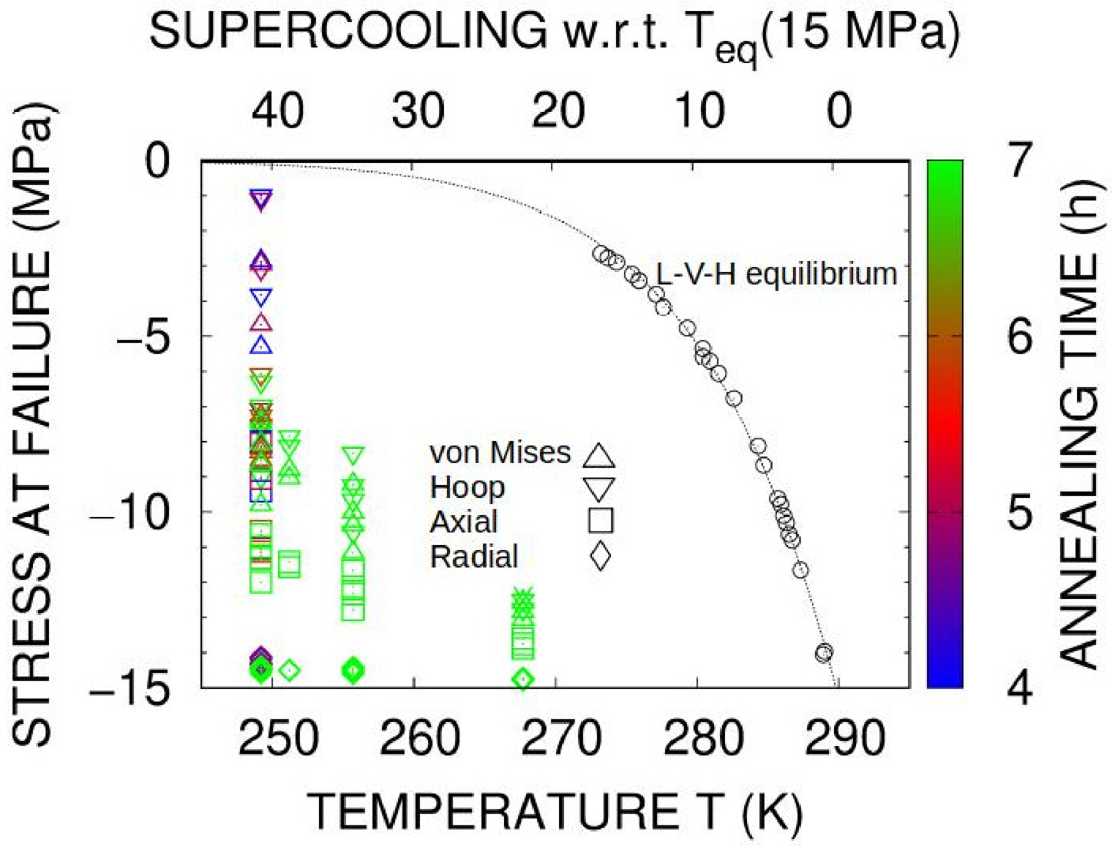

Supplementary Figure 8 | Stress components of the halo at failure, colour coded according to the annealing time. Circles (from Sloan & Koh[10]): water-hydrate-gas equilibrium line (plotted for convenience as $-p$ *vs.* T, so that the domain of stability of the hydrate is below the line); dotted line : Clapeyron-van't Hoff relation as a guide for the eye. This relation also holds for supercooled water at least down to $\approx 260\,\mathrm{K}$ (D. Atig, PhD thesis, Université de Pau Nov. 2019, to be published).

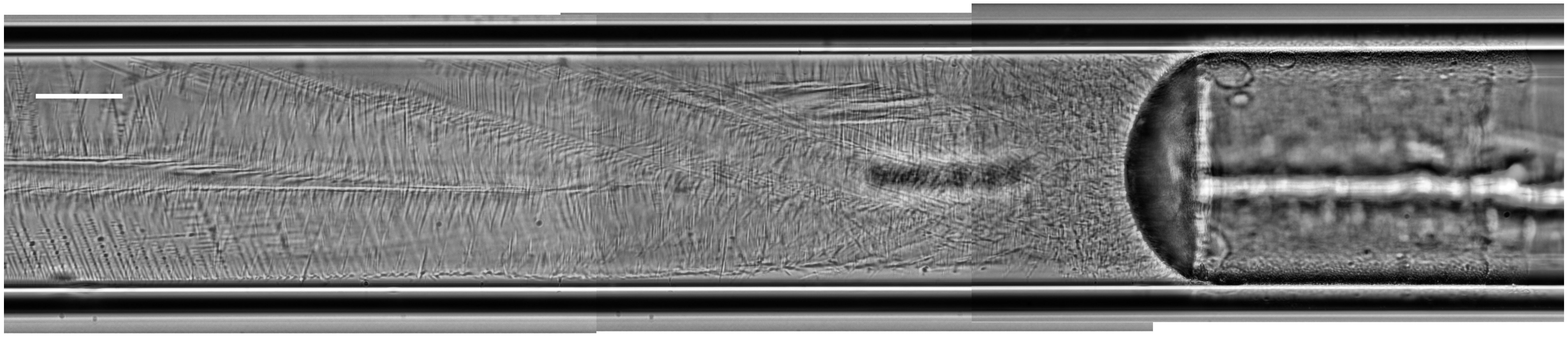

Supplementary Figure 9 | Composite view of transmission micrographs taken $\approx 30\,\mathrm{s}$ after nucleation of methane hydrate as a cap on the former meniscus between water (left) and methane (right, 15 MPa, supercooling $\Delta T = 40.3\,\mathrm{K}$). Needle or feather-like crystals of hydrate propagate left in the water, while a halo of polycrystalline hydrate propagates right, riding over a film of water, see main text. The bright stripe is a jet of water eventually overtaken by the growing halo. Scale bar 100 μm. Image processing: linear contrast stretching and local contrast enhancement (CLAHE in ImageJ 1.52p).

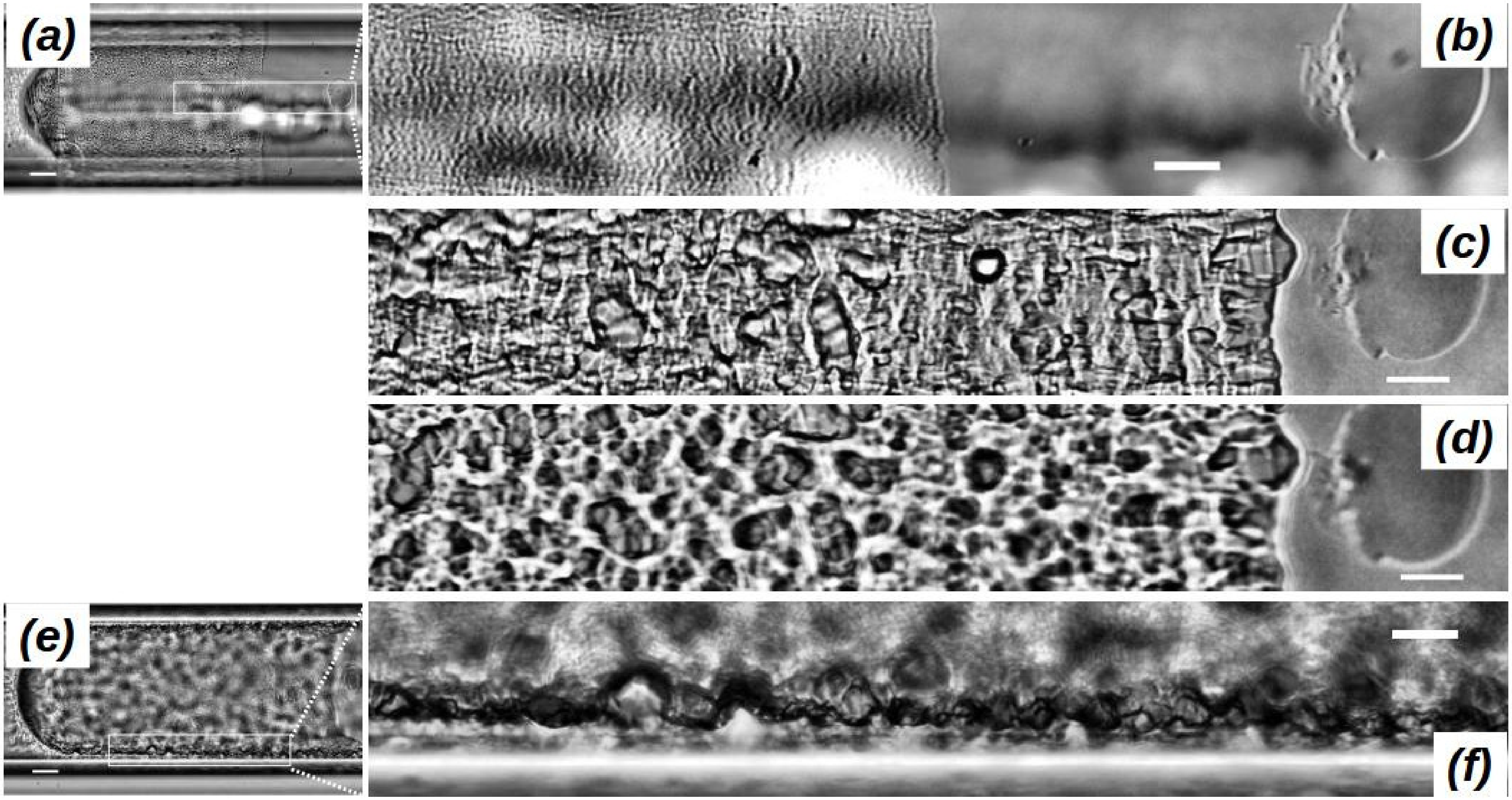

Supplementary Figure 10 | A hydrate shell annealed at pressure $p = 15\,\mathrm{MPa}$ and supercooling $\Delta T = 33.8\,\mathrm{K}$ showing: *(a,b)* Overview and zoomed view of the juvenile shell $\approx 4\,\mathrm{min}$ after nucleation; *(c,d)* Focus on the water and gas sides of the halo at annealing $t_\mathrm{a} \approx 5\,\mathrm{h}$. Striping or ripples in *(b)* are still identifiable with some grain boundaries; *(e)* Cross-section at $t_\mathrm{a} \approx 7\,\mathrm{h}$; *(f)* Zoom of box in *(e)* at the same horizontal scale as *(b–d,f)*. Aberration in *(f)*, due to the curved capillary surface, makes the transverse magnification about 1.35 times the axial magnification. Scale bars: *(a,e)* 50 μm; *(b–d,f)* 20 μm.

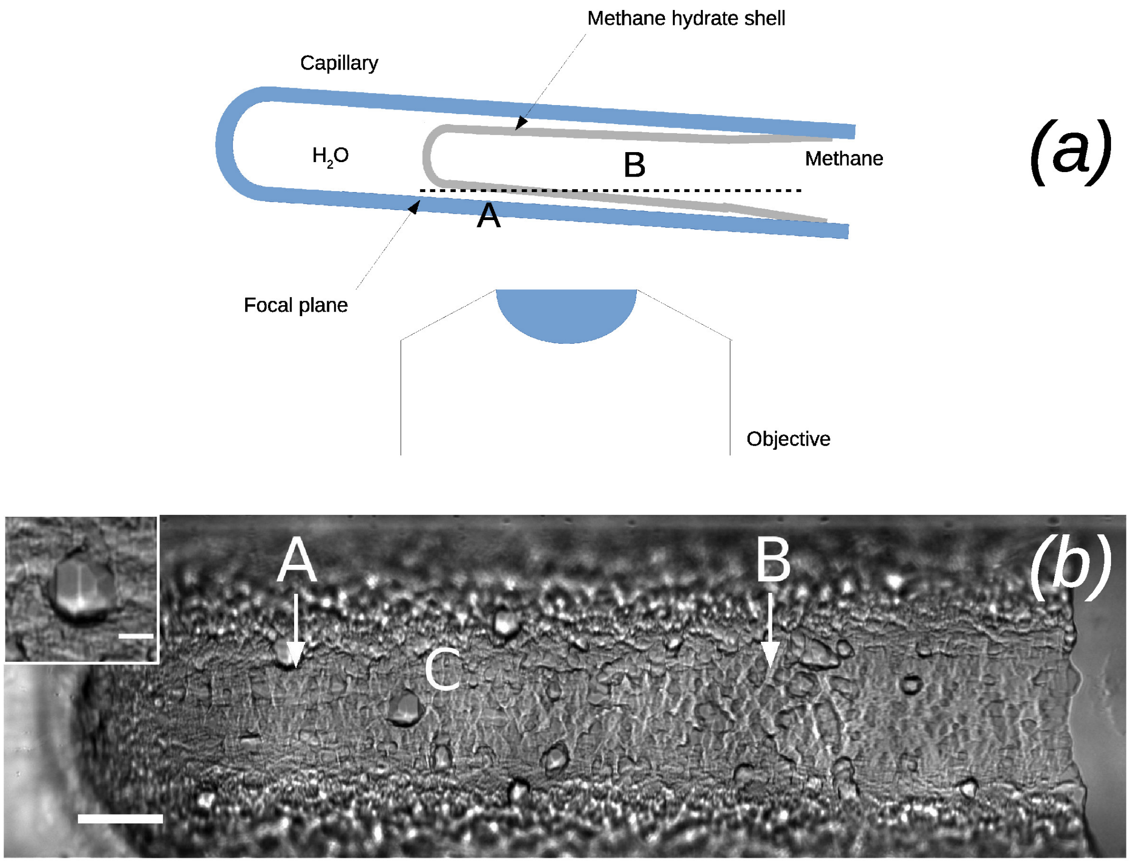

Supplementary Figure 11 | *(a)* Schematic of a capillary set tilted sightly with respect to the focal plane on an inverted microscope, higher on the left than on the right, so the focus is on the under- or water-side of the halo on the left (region A), and on the upper or gas side at B; *(b)* The hydrate shell annealed for $t_a = 6\,\mathrm{h}$ at pressure $p = 15$ MPa and supercooling $\Delta T = 33.8\,\mathrm{K}$ shows a smoother surface on the water side, while crystallites grow into the gas phase. Scale bar 50 μm. The inset (upper left) shows the large faceted crystal at C, scale bar 10 μm.

1 Supplementary References

(1) A.Touil, ACOMPLETER. Ph.D. thesis, Université de Pau et des Pays de l'Adour, Pau, France, 2017.

(2) Bornert, M.; Doumalin, P.; Dupré, J.; Poilâne, C.; Robert, L.; Toussaint, E.; Wattrisse, B. *Exp. Mech.* **2018**, *58*, 33–48.

(3) Williams, T.; Kelley, C.; many others, Gnuplot 5.2: an interactive plotting program. http://gnuplot.sourceforge.net/, 2018.

(4) Landau, L. D.; Lifshitz, E. M. *Theory of Elasticity*, 3rd ed.; Course of Theoretical Physics; Elsevier: Oxford, U.K., 1986; Vol. 7; Chapter 1, p 19.

(5) Makishima, A.; Mackenzie, J. D. *J. Non-Cryst. Solids* **1975**, *17*, 147 – 157, and references therein.

(6) Lehman, R. The mechanical properties of glass. 2019; http://glassproperties.com/references/MechPropHandouts.pdf.

(7) Thermophysical Properties of Fluid Systems" by Eric W. Lemmon, Mark O. McLinden and Daniel G. Friend in NIST Chemistry WebBook, NIST Standard Reference Database Number 69, Eds. P.J. Linstrom and W.G. Mallard, National Institute of Standards and Technology, Gaithersburg MD, 20899, https://doi.org/10.18434/T4D303, (retrieved December 27, 2019).

(8) Kell, G. S. *Journal of Chemical & Engineering Data* **1975**, *20*, 97–105.

(9) Souder, W.; Hidnert, P. *Measurements of the thermal expansion of fused silica*; 1926; Bureau of Standards, Government Printing Office,Washington, D.C.

(10) Sloan, E. D.; Koh, C. A. *Clathrate Hydrates of Natural Gases*, 3rd ed.; Chemical Industries; CRC Press: Boca Raton, FL, 2008; Vol. 119.
