# Supplementary material for: Contactless probing of polycrystalline methane hydrate at pore scale suggests weaker tensile properties than thought
Source: Nat Commun. 2020 Jul 6;11:3379. doi: 10.1038/s41467-020-16628-4 (PMC7338411; doi:10.1038/s41467-020-16628-4)
Supplement: Supplementary file 3 — Description of Additional Supplementary Files [file 41467_2020_16628_MOESM3_ESM.pdf]

Description of Additional Supplementary Files

File name: Supplementary Movie 1
Description: All stages of a monotonic tensile test under strong supercooling, from the formation of methane hydrate to its brittle failure.

File name: Supplementary Movie 2
Description: An illustration of ductile failure under mild supercooling.
